# A Meta-Analysis Reveals Opposite Effects of Biotic and Abiotic Stresses on Transcript Levels of Arabidopsis Intracellular Immune Receptor Genes

**DOI:** 10.3389/fpls.2021.625729

**Published:** 2021-03-04

**Authors:** Leiyun Yang, Zhixue Wang, Jian Hua

**Affiliations:** Plant Biology Section, School of Integrative Plant Science, Cornell University, Ithaca, NY, United States

**Keywords:** NLR, biotic stress, abiotic stress, intracellular immune receptor, Arabidopsis

## Abstract

Plant intracellular immune receptor NLR (nucleotide-binding leucine-rich repeat) proteins sense the presence of pathogens and trigger strong and robust immune responses. NLR genes are known to be tightly controlled at the protein level, but little is known about their dynamics at the transcript level. In this study, we presented a meta-analysis of transcript dynamics of all 207 NLR genes in the Col-0 accession of *Arabidopsis thaliana* under various biotic and abiotic stresses based on 88 publicly available RNA sequencing datasets from 27 independent studies. We find that about two thirds of the NLR genes are generally induced by pathogens, immune elicitors, or salicylic acid (SA), suggesting that transcriptional induction of NLR genes might be an important mechanism in plant immunity regulation. By contrast, NLR genes induced by biotic stresses are often repressed by abscisic acid, high temperature and drought, suggesting that transcriptional regulation of NLR genes might be important for interaction between abiotic and biotic stress responses. In addition, pathogen-induced expression of some NLR genes are dependent on SA induction. Interestingly, a small group of NLR genes are repressed under certain biotic stress treatments, suggesting an unconventional function of this group of NLRs. This meta-analysis thus reveals the transcript dynamics of NLR genes under biotic and abiotic stress conditions and suggests a contribution of NLR transcript regulation to plant immunity as well as interactions between abiotic and biotic stress responses.

## Introduction

Plants in nature are constantly challenged by a variety of environmental stresses including pathogen attacks. In order to fend off pathogens, plants utilize cell-surface receptors and intracellular immune receptors to sense the presence of microbes (Wang et al., 2020). The recognition of pathogens by immune receptors triggers a series of immune responses such as reactive oxygen species burst, Ca^2+^ influx, accumulation of salicylic acid (SA), and transcriptional reprograming (Tsuda and Katagiri, 2010; Buscaill and Rivas, 2014). Transcriptional upregulation of defense genes and reduction of growth-related genes are critical for a successful inhibition or blocking of invasion and propagation of pathogens (Lewis et al., 2015). The plant hormone SA is often induced during defense responses, and key enzymes for SA biosynthesis are regulated by a few transcription factors. SAR DEFICIENT 1 (SARD1) and its close homolog CALMODULIN BINDING PROTEIN 60-LIKE G (CBP60g) are recruited directly to the promoter of *ISOCHORISMATE SYNTHASE 1* (*ICS1*), a key SA biosynthesis gene, to promote its transcription after pathogen infection (Zhang et al., 2010; Wang et al., 2011). *ICS1* can also be bound by members of the TEOSINTE BRANCHED 1/CYCLOIDEA/PCF (TCP) transcription factor family with TCP8 and TCP9 exhibiting the strongest *ICS1*-promoter binding activity (Wang et al., 2015). In addition to positive regulators, several negative regulators have been identified in regulating *ICS1* transcription, and they include ETHYLENE INSENSITIVE 3 (EIN3), EIN3-Like 1 (EIL1) (Chen et al., 2009), and NAC (NAM/ATAF1, ATAF2/CUC2) 19 (Zheng et al., 2012). Interestingly, TCPs interact with SARD1 and NAC019, and some TCPs are induced while others are repressed after pathogen infection (Wang et al., 2015). This suggests that a high-order transcription factor complex is orchestrating and fine-tuning *ICS1* transcription in response to pathogen infection.

Much less is known about the transcript control of plant intracellular immune receptor genes or NLR (nucleotide-binding leucine-rich repeat) genes during defense responses. Plant NLR proteins consist of highly conserved central nucleotide-binding site (NBS), C-terminal leucine-rich repeats (LRRs), and variable N-termini. The N-terminal domain divides NLRs into three groups: toll-interleukin 1 receptor-NLR (TNL), coiled-coil-NLR (CNL), and RPW8-type CC-NLR (RNL) (Monteiro and Nishimura, 2018). They constitute one of the biggest gene families in plants and exhibit great genetic diversity within and among plant species (Van de Weyer et al., 2019; Kourelis and Kamoun, 2020). In the Col-0 accession of *Arabidopsis thaliana*, 207 NLR and NLR-like genes are identified by sequence homology (Meyers et al., 2003) and 51 of them are experimentally validated by a measurable function in disease resistance, defense responses, or autoimmunity (Kourelis and Kamoun, 2020). Some NLRs work as functional singletons that contain both pathogen detection (sensor) and immune signaling (helper) functions, whereas others work either as a sensor or helper in pairs to initiate responses through a complex signaling network (Adachi et al., 2019). The helper NLRs are important signaling hubs for a variety of sensor NLRs (Jubic et al., 2019) and they are encoded by two gene families, *ACTIVATED DISEASE RESISTANCE 1* (*ADR1*) and *N REQUIRED GENE 1* (*NRG1*) in *A. thaliana* (Bonardi et al., 2011; Castel et al., 2019; Lapin et al., 2019; Wu et al., 2019; Saile et al., 2020). It has long been established that NLR protein activity is tightly controlled. Recognition of effectors directly or indirectly causes conformation changes in NLR protein from ADP binding to ATP binding and triggers downstream defense responses (Qi and Innes, 2013; Burdett et al., 2019). Recent reports of the structures of a CNL protein ZAR1 (Wan et al., 2019) and two TNL proteins Roq1 (Martin et al., 2020) and RPP1 (Ma et al., 2020) reveal that recognition of effectors by NLRs triggers oligomerization-dependent NLR activation and downstream immune responses. Whether or not NLR genes are induced during plant pathogen interaction has not been extensively investigated.

Most NLR genes are thought to be expressed at low levels under non-pathogenic conditions (Tan et al., 2007) because constitutive activation of NLR genes often leads to plant dwarfism and sometimes lethality (Gou and Hua, 2012; van Wersch et al., 2016; Wan et al., 2020). However, NLR genes need to be expressed at proper levels to initiate plant immune responses upon pathogen attack (Mohr et al., 2010). Fine control of NLR transcript level under changing environment is therefore potentially critical for balancing defense and growth.

Environmental factors other than pathogens also have a large influence on plant immunity (Hua, 2013; Cheng et al., 2019; Saijo and Loo, 2020). High temperature (Yang and Hua, 2004; Wang et al., 2009; Kim et al., 2010) and high humidity (Jambunathan et al., 2001; Panchal et al., 2016) are often associated with decreased disease resistance while low temperature (Huang et al., 2010; Yang et al., 2010; Kim et al., 2017) and high light (Mühlenbock et al., 2008) are often associated with high disease resistance. The intersection of abiotic factors with immunity could happen at multiple points, and NLR proteins likely occupy key intersection points. High temperature inhibits nuclear accumulation of NLR proteins SNC1 and RPP4, leading to the repression of their induced immune responses at elevated temperature (Zhu et al., 2010; Mang et al., 2012). However, whether or not abiotic stresses impact plant immunity through the regulation of NLR transcription is not thoroughly investigated.

Upregulation of NLR gene expression, in addition to their protein activity activation, has been linked to autoimmunity where immune responses are activated under normal non-pathogenic conditions often leading to spontaneous cell death and dwarfism (van Wersch et al., 2016; Wu et al., 2020). For instance, the immune response in the *hos15-4* mutant defective in histone deacetylation is hyper-activated, which is partially dependent on a NLR gene *SUPPRESSOR OF npr1, CONSTITUTIVE 1* (*SNC1*) (Yang et al., 2020). Additionally, about one third of total NLR genes are upregulated in the *hos15-4* mutant (Yang et al., 2020), suggesting a contribution of NLR activation to autoimmunity. Autoimmune mutants often have increased SA accumulation and spontaneous cell death, which are also highly related to NLR function. For instance, the *acd6-1* mutant is a gain-of-function mutant with increased disease resistance to *Pseudomonas syringae*, and the amount of SA in this mutant is positively correlated with the degree of disease resistance and defense gene expression (Lu et al., 2009). Similarly, the autoimmune mutant *ssi2-1* defective in a stearoyl-ACP desaturase accumulates a high level of SA under normal growth conditions (Shah et al., 2001). The *bak1-4 serk4-1* double mutant defective in two receptor kinases coding *BAK1* and *SERK4* exhibited spontaneous cell death and other autoimmune phenotypes (de Oliveira et al., 2016). However, comprehensive analysis of NLR gene expression in these autoimmune mutants is still lacking.

In this meta-analysis, we systematically analyzed the transcript dynamics of all 207 NLR genes in the Col-0 accession of *A. thaliana* under various biotic and abiotic stresses as well as in autoimmune mutants based on 88 RNA sequencing (RNAseq) datasets from 27 independent studies. We found that about 146 NLR genes were generally induced by pathogens and defense elicitors but repressed by abscisic acid, heat, and drought. The expression pattern of NLR genes in autoimmune mutants was very similar to that in response to pathogen infection. Among the 131 NLR genes induced by pathogens, 87 NLR genes are induced and 44 NLR genes are not induced by SA or its analog BTH. Additionally, 26 NLR genes were repressed under biotic stress conditions. Therefore, this meta-analysis illustrates dynamics of transcript abundance of NLR genes under different conditions, which provides a foundation for further understanding of NLR gene regulation in plant immunity as well as interactions between abiotic and biotic stress responses.

## Methods

### Selection of RNAseq Data for the NLR Expression Study

In this meta-analysis, we utilized publicly available RNAseq data to investigate the transcript dynamics of all 207 NLR genes in the Col-0 accession of *A. thaliana* in response to biotic and abiotic stresses as well as in autoimmune mutants. These NLR or NLR-like genes were listed in previous studies (Meyers et al., 2003; Yang et al., 2020). We also included in the analysis several key SA-related genes because SA signaling is closely linked with NLR activation (Shirano et al., 2002; Xiao et al., 2003; Yang and Hua, 2004). These SA-related genes include SA biosynthesis genes (*ICS1, ICS2, PBS3, EPS1, PAL1, PAL2, PAL3*, and *PAL4*), two master *ICS1* transcription factors *CBP60g* and *SARD1*, and three *EDS1* family genes (*EDS1, PAD4*, and *SAG101*) that mediate SA and NLR signaling, as well as *EDS5* encoding a transporter for SA precursor. Also included are biosynthesis genes of another defense mediator N-Hydroxypipecolic Acid (NHP), *ALD1, SARD4*, and *FMO1*, which are coordinately regulated by the SA regulators SARD1 and CBP60g (Huang et al., 2020).

All the datasets in this meta-analysis were collected from peer-reviewed publications published before August 2020 (Supplementary Data 1). We searched literature *via* Google Scholar for RNAseq data of plants grown under various conditions using the keywords “transcriptome,” “RNA sequencing,” “biotic stress (pathogen infection; SA; flg22; chitin),” “abiotic stress (low temperature; cold; heat; high temperature; salt stress; ABA; drought),” and “autoimmunity.” The datasets used in the meta-analysis were selected based on the following criteria: datasets were performed on leaves or seedlings of Col-0 accessions; differentially expressed genes (DEGs) were listed in the publication or processed reads count for each gene (“reads count-only” datasets) were available in NCBI GEO database. We directly extracted the differentially expressed NLR genes, SA- and NHP-related genes from references if DEGs were listed in the references, and most of these studies used the same criteria (*p* or FDR < 0.05, FC ≥ 2). Otherwise, “reads count-only” datasets were first downloaded from NCBI GEO under the accession numbers provided in references and then DEGs were extracted by in-house pipeline in R with edgeR package. Genes with *p* value < 0.05 were defined as DEGs by the treatment. Because increase of the transcripts of NLR genes is often not very large (sometimes less than 0.5-fold change) after pathogen infection (Zou et al., 2014; Yang et al., 2020) and with or without fold change (FC) does not change the relative numbers of upregulated and downregulated DEGs, we used FDR criteria without an additional FC criterion to allow more sensitive capture of early induction of NLR genes.

RNAseq data with biotic and abiotic treatments as well as RNAseq data for autoimmune mutants were selected. For biotic stresses, we selected infection by representative strains of bacterial pathogen *P. syringae* pv. *tomato* (*Pst*) (*Pst* DC3000, *Pst* DC3000 AvrRps4, *Pst* DC3000 AvrRpm1, *Pst* DC3000 AvrRpt2, and *Pst* DC3000 cor-), fungal pathogen *Botrytis cinerea*, immunity elicitors flg22 and chitin, as well as SA and its functional analog BTH. For abiotic stresses, treatments by ABA, temperature, drought, and salt were included. When data were available, we included at least two independent datasets of the same treatment to increase data confidence. Also included are autoimmune mutants including *hos15-4, acd6-1, ssi2-1*, and *bak1-4 serk4-1*. In total, 88 RNAseq datasets from 27 independent studies were analyzed, including 57 datasets in response to biotic factors, 26 datasets to abiotic factors, and 5 datasets in autoimmune mutants (Table 1). The growth conditions and treatments used in the 27 independent studies are listed in Supplementary Data 1.

**Table 1:** The number of induced and repressed NLR genes extracted from the RNAseq datasets used in this study.

	**Treatments**	**Up**	**Down**	**Total DEGs**	**References**	**Source**	**Cutoffs**
**Biotic stresses**	*Pst* DC3000, 1 hpi	2	4	22	Howard et al., 2013	Directly from the article	*p* ≤ 0.1
	*Pst* DC3000, 6 hpi	9	3	646	Howard et al., 2013	Directly from the article	*p* ≤ 0.1
	*Pst* DC3000, 12 hpi	7	15	1,905	Howard et al., 2013	Directly from the article	*p* ≤ 0.1
	*Pst* DC3000, 24 hpi	20	20	7,254	Yang et al., 2017	GSE90071	*p* < 0.05
	*Pst* DC3000, 1 hpi	3	1	316	Mine et al., 2018	GSE88798	*p* < 0.05
	*Pst* DC3000, 2 hpi	0	0	107	Mine et al., 2018	GSE88798	*p* < 0.05
	*Pst* DC3000, 3 hpi	2	0	201	Mine et al., 2018	GSE88798	*p* < 0.05
	*Pst* DC3000, 4 hpi	3	0	335	Mine et al., 2018	GSE88798	*p* < 0.05
	*Pst* DC3000, 6 hpi	0	1	191	Mine et al., 2018	GSE88798	*p* < 0.05
	*Pst* DC3000, 9 hpi	26	0	1,022	Mine et al., 2018	GSE88798	*p* < 0.05
	*Pst* DC3000, 12 hpi	15	2	2,089	Mine et al., 2018	GSE88798	*p* < 0.05
	*Pst* DC3000, 16 hpi	40	7	5,624	Mine et al., 2018	GSE88798	*p* < 0.05
	*Pst* DC3000, 20 hpi	77	11	8,897	Mine et al., 2018	GSE88798	*p* < 0.05
	*Pst* DC3000, 24 hpi	81	12	9,388	Mine et al., 2018	GSE88798	*p* < 0.05
	*Pst* DC3000 AvrRpm1, 1 hpi	1	14	710	Mine et al., 2018	GSE88798	*p* < 0.05
	*Pst* DC3000 AvrRpm1, 3 hpi	15	2	1,638	Mine et al., 2018	GSE88798	*p* < 0.05
	*Pst* DC3000 AvrRpm1, 4 hpi	51	7	6,002	Mine et al., 2018	GSE88798	*p* < 0.05
	*Pst* DC3000 AvrRpm1, 6 hpi	50	16	8,857	Mine et al., 2018	GSE88798	*p* < 0.05
	*Pst* DC3000 AvrRpm1, 9 hpi	49	14	7,433	Mine et al., 2018	GSE88798	*p* < 0.05
	*Pst* DC3000 AvrRpm1, 12 hpi	61	13	7,977	Mine et al., 2018	GSE88798	*p* < 0.05
	*Pst* DC3000 AvrRpm1, 16 hpi	37	15	7,677	Mine et al., 2018	GSE88798	*p* < 0.05
	*Pst* DC3000 AvrRpm1, 24 hpi	71	4	6,681	Mine et al., 2018	GSE88798	*p* < 0.05
	*Pst* DC3000 AvrRpm1, 3 day, 10h after dawn	45	2	4,389	Schwachtje et al., 2018	GSE101839	*p* < 0.05
	*Pst* DC3000 AvrRpm1, 3 day, 15h after dawn	25	0	1,942	Schwachtje et al., 2018	GSE101839	*p* < 0.05
	*Pst* DC3000 AvrRpm1, 4 day, 9h after dawn	21	5	3,890	Schwachtje et al., 2018	GSE101839	*p* < 0.05
	*Pst* DC3000 AvrRpt2, 1 hpi	8	2	333	Mine et al., 2018	GSE88798	*p* < 0.05
	*Pst* DC3000 AvrRpt2, 2 hpi	0	2	140	Mine et al., 2018	GSE88798	*p* < 0.05
	*Pst* DC3000 AvrRpt2, 3 hpi	8	0	312	Mine et al., 2018	GSE88798	*p* < 0.05
	*Pst* DC3000 AvrRpt2, 4 hpi	91	5	6,102	Mine et al., 2018	GSE88798	*p* < 0.05
	*Pst* DC3000 AvrRpt2, 6 hpi	79	17	11,305	Mine et al., 2018	GSE88798	*p* < 0.05
	*Pst* DC3000 AvrRpt2, 9 hpi	67	15	9,351	Mine et al., 2018	GSE88798	*p* < 0.05
	*Pst* DC3000 AvrRpt2, 12 hpi	67	15	9,579	Mine et al., 2018	GSE88798	*p* < 0.05
	*Pst* DC3000 AvrRpt2, 16 hpi	49	17	9,414	Mine et al., 2018	GSE88798	*p* < 0.05
	*Pst* DC3000 AvrRpt2, 20 hpi	77	10	10,430	Mine et al., 2018	GSE88798	*p* < 0.05
	*Pst* DC3000 AvrRpt2, 24 hpi	61	4	7,177	Mine et al., 2018	GSE88798	*p* < 0.05
	*Pst* DC3000 AvrRps4, 1 hpi	0	0	901	Howard et al., 2013	Directly from the article	*p* ≤ 0.1
	*Pst* DC3000 AvrRps4, 6 hpi	47	6	2,581	Howard et al., 2013	Directly from the article	*p* ≤ 0.1
	*Pst* DC3000 AvrRps4, 12 hpi	33	1	2,501	Howard et al., 2013	Directly from the article	*p* ≤ 0.1
	*Pst* DC3000 cor-, 24 hpi	20	1	2,411	Yang et al., 2017	GSE90071	*p* < 0.05
	*B. cinerea* B05.10, 6 hpi	0	0	1	Coolen et al., 2016	Directly from the article	FDR < 0.05, FC > 2
	*B. cinerea* B05.10, 12 hpi	0	0	67	Coolen et al., 2016	Directly from the article	FDR < 0.05, FC > 2
	*B. cinerea* B05.10, 18 hpi	12	0	780	Coolen et al., 2016	Directly from the article	FDR < 0.05, FC > 2
	*B. cinerea* B05.10, 24 hpi	13	0	1,974	Coolen et al., 2016	Directly from the article	FDR < 0.05, FC > 2
	*B. cinerea* 2100, 14 hpi	59	42	13,078	Liu et al., 2015	GSE66290	*p* < 0.05
	flg22, 30min, 100 nM	39	0	1,247	Li et al., 2015	GSE63603	*p* < 0.05
	flg22, 30min, 1 μM	65	0	2,253	Bazin et al., 2020	GSE146189	*p* < 0.05
	flg22, 1 h, 1 μM	71	17	9,776	Hillmer et al., 2017	GSE78735	*p* < 0.05
	flg22, 2 h, 1 μM	68	16	10,395	Hillmer et al., 2017	GSE78735	*p* < 0.05
	flg22, 3 h, 1 μM	58	15	10,140	Hillmer et al., 2017	GSE78735	*p* < 0.05
	flg22, 5 h, 1 μM	59	17	9,787	Hillmer et al., 2017	GSE78735	*p* < 0.05
	flg22, 9 h, 1 μM	61	8	8,983	Hillmer et al., 2017	GSE78735	*p* < 0.05
	flg22, 18 h, 1 μM	62	4	7,625	Hillmer et al., 2017	GSE78735	*p* < 0.05
	chitin, 3h, 40 mM	105	2	9,353	Yamada et al., 2016	GSE74955	*p* < 0.05
	SA, 1h, 50 μM	38	0	3,367	Ding et al., 2018	Directly from the article	FDR < 0.05, FC ≥ 2
	BTH, 1 h, 300 μM	59	0	1,577	Yang et al., 2017	GSE90077	*p* < 0.05
	BTH, 5 h, 300 μM	70	1	5,695	Yang et al., 2017	GSE90077	*p* < 0.05
	BTH, 8 h, 300 μM	58	1	4,017	Yang et al., 2017	GSE90077	*p* < 0.05
**Abiotic stresses**	44°C, 1 h	18	55	8,851	Suzuki et al., 2016	GSE72806	*p* < 0.05
	37°C, 6 h	16	92	13,700	Pietzenuk et al., 2016	GSE69077	*p* < 0.05
	37°C, 3 h	7	78	8,615	Zhang et al., 2017	GSE94015	*p* < 0.05
	35°C, 4 h	1	10	1,804	Sewelam et al., 2020	Directly from the article	*p* < 0.05, FC > 2
	10°C, 1 h	24	1	824	Schlaen et al., 2015	GSE63406	*p* < 0.05
	10°C, 24 h	10	48	3,733	Schlaen et al., 2015	GSE63406	*p* < 0.05
	4°C, 3 h	5	1	814	Zhao et al., 2016	Directly from the article	*q* < 0.05, FC ≥ 2
	4°C, 24 h	11	13	3,857	Zhao et al., 2016	Directly from the article	*q* < 0.05, FC ≥ 2
	4°C, 24 h	25	76	14,388	Esteve-Bruna et al., 2020	GSE124812	*p* < 0.05
	ABA, 3 h, 50 μM	9	13	4,442	Weng et al., 2016	GSE65739	*p* < 0.05
	ABA, 6 h, 100 μM	36	27	11,771	Zhan et al., 2015	GSE66737	*p* < 0.05
	ABA, 1 h, 10 μM	1	1	491	Song et al., 2016	GSE80565	*p* < 0.05
	ABA, 4 h, 10 μM	4	8	2,318	Song et al., 2016	GSE80565	*p* < 0.05
	ABA, 8 h, 10 μM	2	10	2,090	Song et al., 2016	GSE80565	*p* < 0.05
	ABA, 12 h, 10 μM	3	12	2,287	Song et al., 2016	GSE80565	*p* < 0.05
	ABA, 24 h, 10 μM	3	11	1,960	Song et al., 2016	GSE80565	*p* < 0.05
	ABA, 36 h, 10 μM	4	16	2,498	Song et al., 2016	GSE80565	*p* < 0.05
	ABA, 60 h, 10 μM	2	18	2,334	Song et al., 2016	GSE80565	*p* < 0.05
	ABA, 3h, 50 μM	24	38	8,527	Zhu et al., 2017	GSE99677	*p* < 0.05
	drought, 5 days	0	0	804	Coolen et al., 2016	Directly from the article	FDR < 0.05, FC > 2
	drought, 6 days	4	33	2,486	Coolen et al., 2016	Directly from the article	FDR < 0.05, FC > 2
	drought, 7 days	2	35	3,219	Coolen et al., 2016	Directly from the article	FDR < 0.05, FC > 2
	low water potential, 96 h	5	31	2,856	Wong et al., 2019	Extracted from the article	*p* < 0.05
	NaCl, 1 h, 150 mM	0	16	958	Suzuki et al., 2016	GSE72806	*p* < 0.05
	NaCl, 7 h, 150 mM	0	0	85	Sewelam et al., 2020	Directly from the article	*p* < 0.05, FC > 2
	NaCl, 24 h, 150 mM	65	56	15,125	Esteve-Bruna et al., 2020	GSE124812	*p* < 0.05
**Autoimmune mutants**	*acd6-1*, 1h after light onset	100	2	8,595	Zhang et al., 2019	GSE115680	*p* < 0.05
	*acd6-1*, 1h after darkness onset	75	1	5,548	Zhang et al., 2019	GSE115680	*p* < 0.05
	*bak1-4 serk4-1*	43	2	3,637	de Oliveira et al., 2016	Directly from the article	FDR < 0.1, FC ≥ 2
	*ssi2-1*	72	22	6,316	Yang et al., 2016	Directly from the article	FDR < 0.001, FC ≥ 2
	*hos15-4/smo1*	74	0	3,512	Yang et al., 2020	Directly from the article	FDR < 0.05

*Shown are the selected RNAseq datasets including 57 biotic stresses, 26 abiotic stresses, and 5 autoimmune mutants. The differentially expressed genes (DEGs) are either directly from the publication or extracted from the deposited data on NCBI GEO website using in-house pipeline. The cutoff for in-house pipeline is p < 0.05 to capture subtle changes of NLR genes. The cutoffs for DEGs from the references are either the same as in the publication or p < 0.05 is used when the whole transcriptome changes are listed (for Wong et al., 2019). “Up” indicates “induced NLRs” while “Down” indicates “repressed NLRs”. “h,” hour; “hpi,” hour(s) post inoculation; “FDR,” false discovery rate; “FC,” fold change*.

### Procedures for Extracting DEGs From “Reads Count-Only” Datasets

Reads count of each treatment or mutant was downloaded from NCBI under GSE accessions provided in the references (Table 1). R/_EDGE_R was used to call DEGs. The cpm (counts per million) values of each gene were calculated using “y <- DGEList(counts =x,group=group)”; “y <- calcNormFactors(y),” and genes with cpm value > 1 in at least two samples were used for DEG analysis using “keep <-rowSums(cpm(y)≥1) ≥2”; “y <-y[keep,].” Then, DEGs were called using “design <-model.matrix(~0+group)”; “y <- estimateGLMCommonDisp(y,design)”; “y <- estimateGLMTrendedDisp(y,design)”; “y <- estimateGLMTagwiseDisp(y,design)”; “fit <-glmFit(y,design)”; “lrt.2vs1 <- glmLRT[fit, contrast=c(-1,1)]”; “top2v1 <- topTags(lrt.2vs1, n=30000)”. For more details, please see https://biohpc.cornell.edu/workshops.aspx.

### Hierarchical Cluster Analysis of NLR Genes and SA- and NHP-Related Genes

The hierarchical cluster analysis was performed using “hclust” function in R program (https://www.r-project.org/). Because some NLR genes have been shown to have feedback regulation with SA, which is tightly connected with NHP, the SA- and NHP-related genes were also included in this analysis. To increase robustness of the analysis, we excluded 15 RNAseq datasets with fewer than 10 differentially expressed NLR genes. To reduce redundancy, one time point with the most drastic changes of NLR genes was selected for each of the time-course treatment. In total, 37 different samples were used for cluster analysis, with 16 biotic stress treatment, 16 abiotic stress treatment, and 5 autoimmune mutants. Briefly, the values of each gene with upregulation, downregulation, and no change were considered as 1, −1, and 0, respectively. The dissimilarity values were calculated with the “dist” function and then used as input for “hclust.” The Ward method was used for hierarchical clustering, because it identified the strongest clustering structure among the four methods (Average, 0.5804108; Single, 0.4357676; Complete, 0.7109366; Ward, 0.897766) analyzed. The clusters were identified with the “cutree” function, and the number of optimal clusters was determined with the Elbow method. Heatmap was generated in Excel. For visualization, NLR genes induced by the treatments or upregulated in the mutants were colored red while genes repressed were colored blue. Genes not expressed or the transcript level not altered were left blank. Among the 207 total NLR genes, 29 NLR genes had no altered expression or were not expressed in response to any of the 37 conditions selected in this analysis (Table 2). None of these genes were experimentally validated and 13 of them did not contain NBS and LRR domains (Table 2). Additionally, 11 of them are no longer considered as valid genes on TAIR (Table 2). Therefore, we excluded these genes from the cluster analysis for clarity.

**Table 2:** Information for the 29 “non-expressed” or “non-altered” NLR genes.

**Locus**	**ID**	**Type**	**EV-NLR**	**Expression**
AT1G57670		TX		Non-altered
AT1G63870		TNL		Non-altered
AT3G51560		TNL		Non-altered
AT3G51570		TNL		Non-altered
AT4G27190		CNL		Non-altered
AT5G17970		TNL		Non-altered
AT5G45200		TNL		Non-altered
AT1G57830		TX		Non-altered
AT4G08450		TNL		Non-altered
AT4G10780		CNL		Non-altered
AT5G49140		TNL		Non-altered
AT1G61105		TX		Non-expressed
AT5G17950		CN		Non-expressed
AT5G45230		TNL		Non-expressed
AT1G60320		TX		Non-expressed
AT2G03030		TX		Non-expressed
AT2G03300	ATTX12	TX		Non-expressed
AT4G04110		TN		Non-expressed
AT1G58842*		CNL		Non-expressed
AT3G25515*		TNL		Non-expressed
AT5G46480*		TN		Non-expressed
AT1G51280*		TX		Non-expressed
AT1G51485*		CNL		Non-expressed
AT2G20145*		TX		Non-expressed
AT4G11345*		TX		Non-expressed
AT4G19923*		TX		Non-expressed
AT4G19926*		TX		Non-expressed
AT4G23513*		TX		Non-expressed
AT4G23516*		TX		Non-expressed

The locus, gene common name (ID), and NLR type of 29 NLR genes not included in the cluster analysis of **Figure 2**. These genes are either not expressed or their transcript levels are not altered in any of the treatment in the RNAseq datasets in **Figure 2**. “non-altered” indicates that the transcripts are neither increased nor decreased. “EV-NLR” means “experimentally validated NLRs” defined by Kourelis and Kamoun (2020). Genes with “*” are not valid genes on TAIR.

## Results

### NLR Genes Are in General Induced Under Biotic Stresses

We tallied NLR genes that have increased or reduced expression respectively for each treatment (Table 1). In 47 out of 57 biotic stress treatments, more NLR genes had increased expression than reduced expression after treatment (Table 1). For instance, 81 NLR genes were induced while 12 were repressed at 24 h post infiltrating with *Pst* DC3000 (Table 1). Also, chitin treatment at 40 mM for 3 h induced 105 NLRs while repressed only 2 NLRs (Table 1). The 10 treatments that did not show more NLR genes induced than repressed were either not reproduced in independent studies or were for the early time point after infection and became to have more induced NLR genes in later time points. Therefore, all biotic treatments analyzed, *Pst* DC3000 strains (virulent, avirulent, or non-virulent), *B. cinerea*, flg22, chitin, and SA, led to more NLR genes having increased gene expression than reduced gene expression.

**Figure 1:**
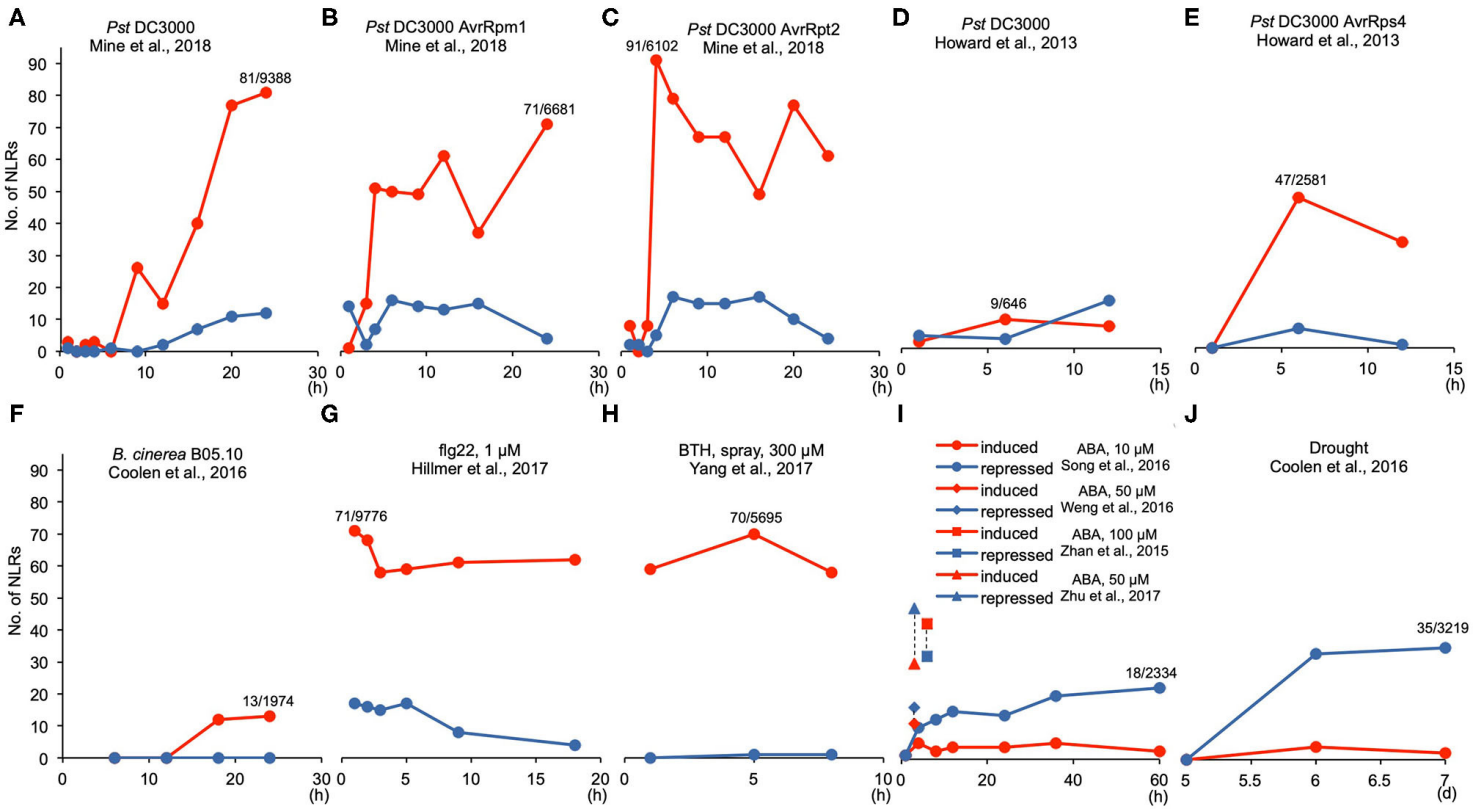
Transcript dynamics of NLR genes during biotic and abiotic stresses. **(A–J)** Line graphs showing the number of induced (red color) and repressed (blue color) NLR genes at each time point for different treatments. The numbers are made based on Table 1. For biotic stress treatments, the maximum number of induced NLR genes and the total DEG number are listed as induced NLR number/total DEG number above the corresponding time point. Similarly for abiotic stress treatments, the maximum number of repressed NLR genes and the corresponding total DEGs are listed. “h” indicates “hours” and “d” means “days”.

We further viewed the transcript dynamics in response to different treatments by plotting the number of increased and reduced NLR genes over time in the same treatment with a study. In general, the number of NLR genes with altered expression increased as the treatment progressed, and this was especially pronounced for the number of NLR genes with increased expression (Figure 1). The study of Mine et al. (2018) contained a set of treatment by various *Pst* DC3000 strains, which allowed us to compare over time and across different strains for DEGs selected by *p* < 0.05. After *Pst* DC3000 infection, fewer than 5 NLR genes had altered expression before 6 hpi (hours post-inoculation) while 40, 77, and 81 NLR genes were induced at 16, 20, and 24 hpi, respectively (Table 1; Figure 1A). By contrast, no more than 13 NLR genes were repressed during all the time points (Table 1; Figure 1A). A similar increase of number of induced NLRs over time was also observed under the infection by avirulent bacterial pathogens *Pst* DC3000 AvrRpm1 and *Pst* DC3000 AvrRpt2. Very few NLRs had altered gene expression before 3 hpi, but more NLR genes had increased expression after 4 hpi (Table 1; Figures 1B,C). A maximum of 71 and 91 NLR genes were induced by *Pst* DC3000 AvrRpm1 and *Pst* DC3000 AvrRpt2, respectively (Table 1; Figures 1B,C). The number of induced NLR genes went up much faster in response to avirulent *Pst* DC3000 strains compared to the virulent *Pst* DC3000. No more than 26 NLR genes were induced in response to *Pst* DC3000 before 16 hpi whereas 51 and 91 NLR genes were induced at 4 hpi by *Pst* DC3000 AvrRpm1 and *Pst* DC3000 AvrRpt2, respectively (Table 1; Figures 1A–C). A similar pattern was also observed in the study of Howard et al. (2013). With DEG selected by *p* ≤ 0.1, no more than 10 NLR genes were induced in response to *Pst* DC3000 at 1, 6, and 12 hpi while *Pst* DC3000 AvrRps4 induced 47 and 33 NLR genes at 6 and 12 hpi, respectively (Table 1; Figures 1D,E). This suggested a faster defense response in incompatible interaction than compatible interaction, as observed earlier in overall transcriptome responses (Tao et al., 2003).

The infection by fungal pathogens also induced the transcript level of some NLR genes. In response to fungal pathogen *B. cinerea* B05.10 strain, 12 and 13 NLR genes were upregulated at 18 and 24 hpi, respectively, and no NLR genes were repressed in the study of Coolen et al. (2016) (Table 1; Figure 1F). In another dataset of infection by the *B. cinerea* 2100 strain, with *p* < 0.05, 59 NLR genes were upregulated and 42 were downregulated at 14 hpi (Table 1). The latter dataset had an unusual high number of DEGs (comprising half of the genome), which might contribute to the high number of NLR genes differentially expressed in this dataset (Table 1). More datasets will be needed for determining the general NLR expression patterns during fungal pathogen infection.

The treatment of flg22, chitin, SA, and BTH all led to more NLR genes with increased expression than with repressed gene expression (Table 1). Using the same DEG selection criteria, the induction apparently occurred earlier than pathogen infection even by avirulent *Pst* DC3000 strains (Table 1; Figures 1G,H). In Hillmer et al. (2017), 1 μM flg22 induced a maximum of 71 NLR genes at 1 h post treatment (Figure 1G) and 65 NLR genes were induced at 30 min post treatment of 1 μM flg22 in another study (Bazin et al., 2020) with the same DEG selection criteria *p* < 0.05 (Table 1). Likewise, 38 NLR genes were induced at 1 h post treatment of 50 μM SA (Table 1). Additionally, no NLR genes were repressed by SA treatment, and only one or two NLR genes were repressed by BTH or chitin (Table 1). The PAMP (Pathogen-Associated Molecular Pattern) signal flg22 at 100 nM and 1 μM treatment did not repress NLR gene expression at early time point (30 min), but repressed at maximum 17 NLR genes at 1 μM, while it induced expression of 39–71 NLRs under all conditions (Table 1). After the early induction with these molecules, the number of NLR gene with increased expression sustained throughout the duration of treatment (Figures 1G,H).

### NLR Genes Are in General Repressed in Response to Heat, ABA, and Drought

Abiotic stresses including high temperature and ABA are found to impact plant immunity through affecting NLR protein localization (Zhu et al., 2010; Mang et al., 2012). In addition, a previous study showed that the variation in NLR gene expression may be under natural section to better adapt to the environment (MacQueen and Bergelson, 2016). Here, we analyzed NLR transcript change in response to abiotic stresses including heat, ABA, and drought and found that these abiotic stresses in general repressed the transcript level of NLR genes (Table 1).

Under three heat shock treatments, 44°C for 1 h, 37°C for 3 or 6 h, and 35°C for 4 h, much more NLR genes were repressed than induced (Table 1). Three-hour 37°C treatment repressed 78 NLR genes and induced 7 NLR genes among a total of 8,615 DEGs (Table 1). Similarly, 1-h 44°C treatment repressed 55 NLR genes and induced 18 NLR genes among 8,851 DEGs (Table 1). Therefore, more NLR genes had decreased transcripts than increased expression, which might contribute to high-temperature inhibition of disease resistance. By contrast, low temperature had a more complex effect on NLR transcript level depending on the duration of cold treatment. At the early period of low-temperature treatment (4°C for 3 h or 10°C for 1 h), no more than 25 NLR genes had altered expression with more genes induced than repressed by low temperature as observed in two independent studies by Zhao et al. (2016) and Schlaen et al. (2015) (Table 1). However, under 24 h of 4 and 10°C treatment, 13 and 48 NLR genes were repressed, along with 11 and 10 NLR genes induced, respectively (Table 1). This was supported by another study (Esteve-Bruna et al., 2020) in which 76 NLR genes were repressed while only 25 NLR genes were induced after 24 h treatment of 4°C (Table 1).

Similar to heat, ABA and drought generally repressed the expression of NLR genes (Table 1; Figures 1I,J). Under 10 μM ABA treatment in Song et al. (2016), more NLR genes were repressed than induced and the number of repressed NLR genes increased as treatment time increased (Table 1; Figure 1I). Similarly, higher concentrations of ABA generally have more NLR genes repressed than induced (Table 1; Figure 1I). Drought treatment, including low water potential treatment, also drastically repressed the transcript levels of NLR genes (Table 1). A total of 33 and 35 NLR genes were repressed under drought treatment of 6 and 7 days, respectively, while fewer than 5 NLRs were induced (Table 1; Figure 1J). Likewise, 31 NLR genes were repressed while only 5 NLR genes were induced among 2,856 DEGs under low water potential treatment for 96 h (Table 1).

NaCl treatment did not appear to cause specific transcript changes of NLR genes (Table 1). NaCl treatment at 150 mM for 1 h (Suzuki et al., 2016) led to 16 NLR genes repressed and no NLR induced with *p* < 0.05. However, NaCl treatment at 150 mM for 7 h from another study (Sewelam et al., 2020) did not alter any NLR gene expression with *p* < 0.05 and FC > 2. A longer treatment time of 24 h (Esteve-Bruna et al., 2020) altered expression of a large number of NLR genes, with 65 upregulated and 56 downregulated. It is worth noting that three quarters of genes in the genome had altered expression in this study (Table 1), and therefore whether or not NaCl has a specific effect on NLR gene expression at this stage of salt stress still needs investigation.

### NLR Genes Are Induced in Autoimmune Mutants

Because activation of NLR genes has been linked to autoimmunity, we examined all NLR genes to determine which and how many were differentially expressed in autoimmune mutants. The results showed that the transcripts of 43–100 NLR genes were increased in all the mutants with fewer than three genes repressed for all but the *ssi2-1* mutant (Table 1). The *acd6-1* mutant had 100 and 75 induced NLR genes at 1 h after light or darkness onset, respectively, and only 1–2 NLR genes were repressed under these conditions with *p* < 0.05 (Table 1). Likewise, the *bak1-4 serk4-1* double mutant had 43-induced NLR genes while only 2 repressed NLR genes with FDR < 0.1 and FC ≥ 2 (Table 1). The *hos15-4* mutant had 74-induced NLR genes, and no NLR genes were repressed in this mutant with FDR < 0.05 (Table 1). Although 22 NLR genes were repressed, the *ssi2-1* mutant had 72-induced NLR genes among 6,316 DEGs under relatively stringent DEG selection criteria of FDR < 0.001 and FC ≥ 2 (Table 1). These analyses suggest that activation of NLR genes is a shared phenomenon for autoimmune mutants.

### Expression of Majority of NLR Genes Are Induced by Biotic Stresses and Repressed by Abiotic Stresses

**Figure 2:**
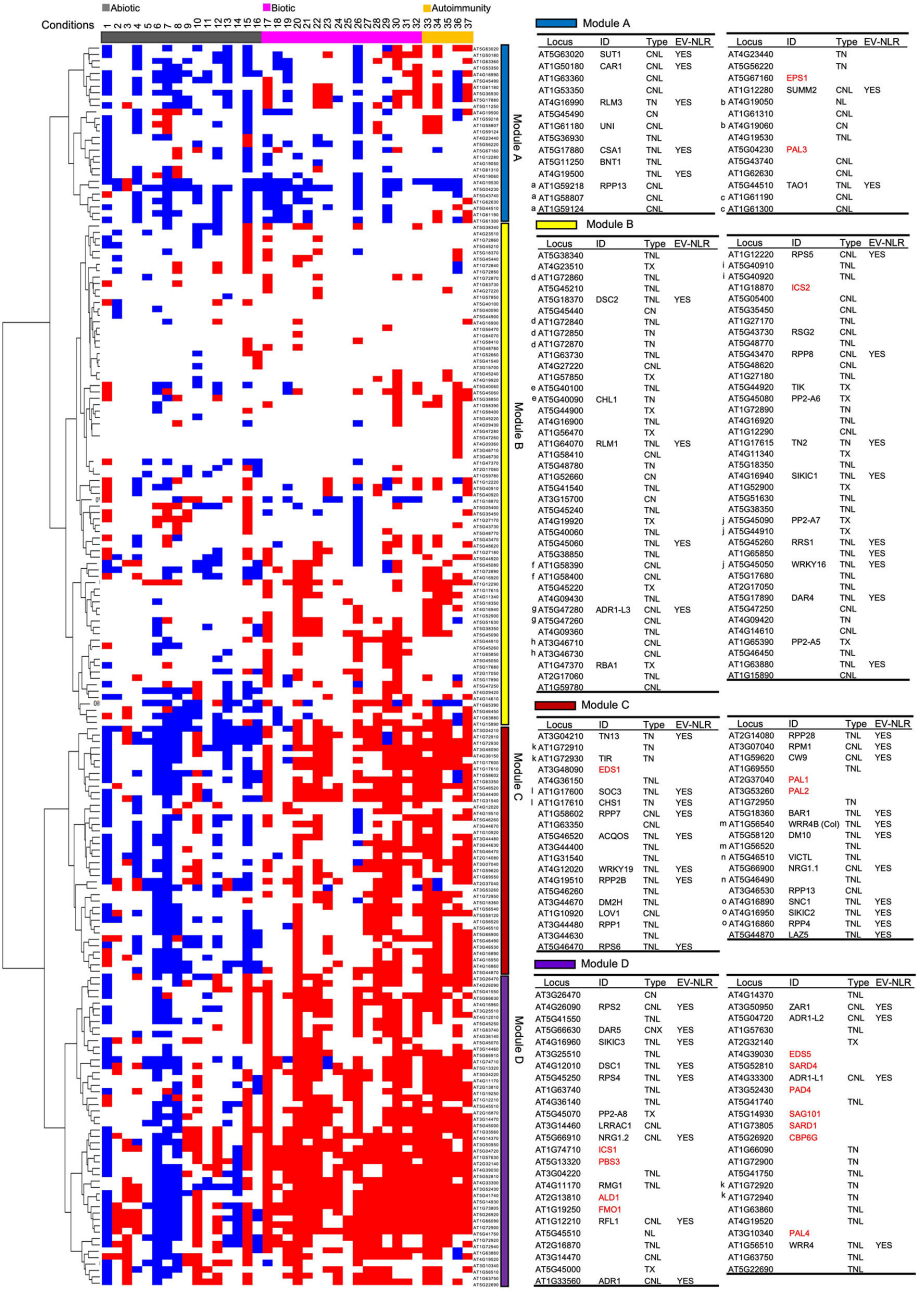
Expression profile of 178 NLR genes as well as 17 SA- and NHP-related genes under stress conditions and in autoimmune mutants. Cluster display of 178 NLR or NLR-like genes along with 17 SA- and NHP-related genes by their transcript levels in 37 conditions. Gene induction is marked as a red box while gene repression is marked as a blue box. Expression not altered are left as blank. Modules A, B, C, and D are discussed in the text. The detailed information of the genes from each module is shown in the right tables, including gene ID number on TAIR website, common name, NLR type, and whether genes are experimentally validated or not. In the tables, 17 SA- and NHP-related genes are colored red. “EV-NLR” means “experimentally validated NLRs” defined by Kourelis and Kamoun (2020). The letter on the left of a gene indicates that this gene is co-regulated with at least another gene in the respective gene cluster and the same letter before the gene ID indicates genes are in the same gene cluster on the chromosome. The 37 conditions are numbered and they are (1) 4°C, 24 h; (2) 4°C, 24 h; (3) 10°C, 1 h; (4) 10°C, 24 h; (5) 35°C, 4 h; (6) 37°C, 3 h; (7) 37°C, 6 h; (8) 44°C, 1 h; (9) ABA, 3 h, 50 μM; (10) ABA, 6 h, 100 μM; (11) ABA, 60 h, 10 μM; (12) ABA, 3 h, 50 μM; (13) drought, 7 days; (14) NaCl, 1 h, 150 mM; (15) NaCl, 24 h, 150 mM; (16) low water potential, −0.7 Mpa, 96 h; (17) *Pst* DC3000, 24 hpi, OD_600_ of 0.001; (18) *Pst* DC3000, 12 hpi, 107 cfu/ml; (19) *Pst* DC3000, 24 hpi, OD_600_ of 0.2; (20) *Pst* DC3000 AvrRpt2, 4 hpi, infiltration, OD_600_ of 0.001; (21) *Pst* DC3000 AvrRps4, 6 hpi, 107 cfu/ml; (22) *Pst* DC3000 AvrRpm1, 24 hpi, OD_600_ of 0.001; (23) *Pst* DC3000 AvrRpm1, 3 day, 10 h after dawn, OD_600_ of 0.02; (24) *Pst* DC3000 cor-, 24 hpi, OD_600_ of 0.2; (25) *B. cinerea* B05.10, 24 hpi, 5 μl 1 ×10^5^ spores/ml; (26) *B. cinerea* 2100, 14 hpi, 2 μl 2.5 ×10^5^ spores/ml; (27) flg22, 30 min, 100 nM; (28) flg22, 30 min, 1 μM; (29) flg22, 1 h, 1 μM; (30) chitin, 3 h, 40 mM; (31) SA, 1 h, 50 μM; (32) BTH, 5 h, spray, 300 μM; (33) *acd6-1*, ZT13, 1 h after darkness onset; (34) *acd6-1*, ZT1, 1 h after light onset; (35) *bak1-4 serk4-1*; (36) *ssi2-1*; (37) *hos15-4/smo1*. References and detailed information for these conditions (1–37) are in Supplementary Data 1. Abiotic stresses are colored gray, biotic stresses are colored magenta, and autoimmune mutants are colored orange. “h” indicates “hour” and “hpi” means “hour(s) post inoculation”.

To reveal potential gene network among all NLR genes, as well as SA- and NHP-related genes, we did a hierarchical cluster analysis of these genes based on their induction or repression under selected stress conditions and in autoimmune mutants (see *Methods* for details). Expression pattern was displayed for clustered NLR genes with grouped abiotic treatment, biotic treatment, and autoimmune mutants. Overall pattern from the cluster analysis revealed that abiotic and biotic stresses in general had opposite effects on expression of half of the NLR gene expression (Figure 2). Based on the expression pattern, NLR genes could be grouped into four modules A–D (Figure 2). Modules C and D comprised about half of the NLR genes and they were more similar to each other than to modules A and B. NLR genes in modules C and D were generally induced by biotic stresses and repressed by abiotic stresses, but they slightly differed in the extent of expressional changes.

Modules C and D contained 74 NLR genes, which were induced by most of the biotic stresses and repressed by most of the abiotic stresses (Figure 2). NLR genes in module D had more induction under biotic stresses while less repression under abiotic stresses as compared to NLR genes in module C. Fifty-five of them contained a TIR domain, suggesting that TNLs were more likely to respond to biotic and abiotic stresses than CNLs. Additionally, 31 out of the 51 experimentally validated NLR genes were in these two modules. For instance, *RPM1* in module C was induced by both *Pst* DC3000 and *Pst* DC3000 AvrRpm1 at 24 hpi while it was repressed at 3 h or 6 h at 37°C. Notably, five major helper NLR genes *ADR1, ADR1-L1, ADR1-L2, NRG1.1*, and *NRG1.2* were in these two modules (Figure 2). The drastic distinct expression changes of NLR genes under biotic and abiotic stresses suggested that transcriptional regulation of NLR genes might be an important mechanism for plants to cope with different environmental stresses.

Module B had 78 NLR genes including 13 experimentally validated NLRs, and 24 of them did not contain the LRR domain (Figure 2). NLR genes in this module were generally induced under a small number of biotic stress treatments and repressed under a small number of abiotic conditions, although some of them were induced under some abiotic conditions and repressed under certain biotic stresses (Figure 2). These NLR genes might have specificity in response to biotic and abiotic stresses. Alternatively, the expression level was too small to be detected in the RNAseq. Consequently, module B had a more heterogeneous pattern than modules C and D, which exhibited drastic expression changes of NLR genes under stress conditions.

### A Small Number of NLR Genes Are Repressed by Biotic Stresses

Module A contained 26 NLR genes that were generally repressed under both biotic and abiotic stresses and in autoimmune mutants, with abiotic stresses more often repressing their expression than biotic stresses (Figure 2). Sixteen of them contained the CC domain, and 7 were experimentally validated NLR genes including *SUMM2* (*SUPPRESSOR OF MKK1 MKK2 2*) and *TAO1* (*TARGET OF AVRB OPERATION 1*). For instance, the transcript level of a CNL *SUMM2* was decreased by 150 mM NaCl treatment at 24 h and 50 μM ABA treatment at 3 h. At the same time, it was repressed by biotic stresses including *B. cinerea, Pst* DC3000, and *Pst* DC3000 AvrRpt2. This might suggest a unique mode of action of CNLs under certain pathogenic conditions and further studies are needed to explore the biological relevance of this group of NLR genes in response to biotic and abiotic stresses.

### A Large Group of NLR Genes Have Similar Expression Pattern With SA- and NHP-Related Genes

All the 17 SA- and NHP-related genes except for *ICS2, EPS1*, and *PAL3* were in module C or D, and 11 of them were in module D, which had stronger induction of NLR genes under biotic stresses (Figure 2). Specifically, *PAD4, SAG101, CBP60g*, and *SARD1* were in the same small subclade with *ADR1-L1, AT5G41740, AT5G41750, AT1G66090*, and *AT1G72900*. *EDS5* and *SARD4* were in the same subclade with *ADR1, ADR1-L2, ZAR1, AT4G14370, AT1G57630*, and *AT2G32140*. The *PAD4* subclade and the *EDS5* subclade were close to each other in the cluster. *ICS1* and *PBS3* were in another subclade, and so were *ALD1* and *FMO1*. The SA biosynthesis genes *PAL1* and *PAL2* were in a subclade of module C. *EDS1*, a gene functionally related to *PAD4*, was not in the *PAD4* subclade but was in the same subclade with two experimentally validated NLR genes *AT1G17600* (*SOC3*) and *AT1G17610* (*CHS1*). By contrast, *EPS1* and *PAL3* were in module A and *ICS2* was in module B (Figure 2). In addition, the expression patterns of *ADR1s* (*ADR1, ADR-L1*, and *ADR-L2*) were more similar to SA-related genes as compared to that of *NRG1s* (*NRG1.1, NRG1.2*) (Figure 2). This was consistent with previous studies in which the *ADR1* gene family regulates immunity through regulation of SA accumulation and subsequent activation of SA-dependent responses while the *NRG1* gene family is not involved in SA regulation (Bonardi et al., 2011; Castel et al., 2019). Notably, 31 out of 38 SA-induced and 57 out of 78 BTH-induced NLR genes were also in modules C and D. These results suggest that SA and biotic stresses mostly activated a similar set of NLR genes.

### Assessment of the Contribution of SA to NLR Induction Under Biotic Stresses

**Figure 3:**
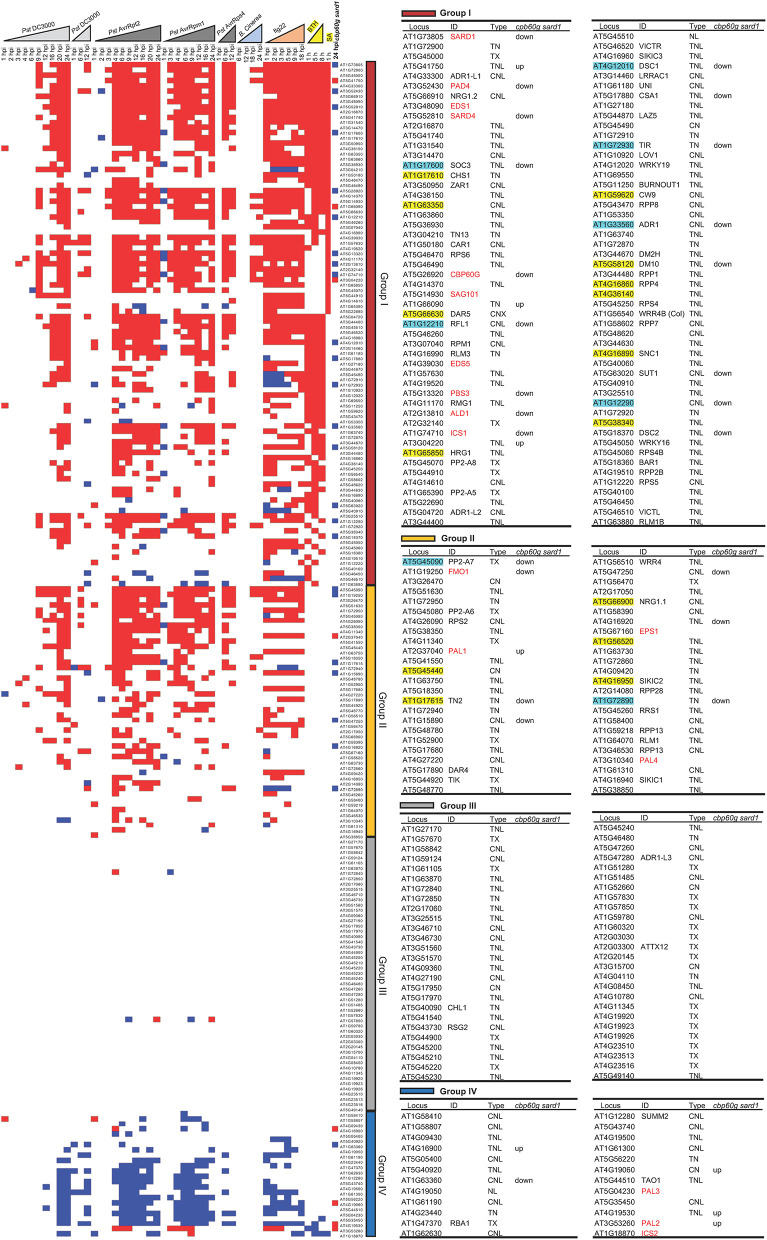
Expression profile of 207 NLR genes as well as 17 SA- and NHP-related genes under time-course biotic stress treatment. Expression levels of 207 NLR or NLR-like genes and 17 SA- and NHP-related genes during the time course of biotic stress treatments that are marked as triangles (light gray for *Pst* DC3000; dark gray for avirulent pathogens including *Pst* DC3000 AvrRpt2, *Pst* DC3000 AvrRpm1, and *Pst* DC3000 AvrRps4; light blue for *B. cinerea*; orange for flg22; and yellow for BTH). The genes are ordered by their induction by SA and BTH (first ordered by the induction by SA, then ordered by the induction by BTH), from induced (indicated by red square), to non-alteration (blank) and repressed (blue square) from top to bottom. These genes are grouped into I (dark red), II (orange), III (gray), and IV (dark blue) four groups based on the transcript changes under biotic stresses as discussed in *Results*. NLR genes that exhibited a reduced expression or an increased expression in the *cbp60g sard1* mutant compared to the wild type at 24 hpi after *P. syringae* pv *maculicola* ES4326 infection are displayed as blue square or red square, respectively, in the last column of the expression heatmap. The 17 SA- and NHP-related genes are colored red. The 15 NLR genes dependent on SA and the 8 NLR genes of which full induction is dependent on SA are highlighted with yellow and light blue, respectively. The detailed information of the genes from each group is shown in the right tables, including gene ID number on TAIR website, common name, NLR type, and whether genes are upregulated (up) or downregulated (down) in the *cbp60g sard1* mutant compared to wild-type plant at 24 hpi after *P. syringae* pv *maculicola* ES4326 infection.

We further investigated the contribution of SA to NLR induction under biotic stresses. Datasets with time-course biotic stress treatments along with SA and BTH treatments were used for plotting the transcript dynamics of NLR genes. NLR genes were categorized into four major groups. Group I contained 90 NLR genes that are induced by SA or BTH, and 87 NLR genes among them were also induced by at least one of the biotic stresses (Figure 3). Most NLR genes are induced by pathogens at 4 hpi when SA-related genes, including *PAD4, EDS1, SARD1, CBP60g, SAG101, EDS5, PBS3*, and *ICS1*, were induced (Figure 3). SA may potentially be involved in the induction of some NLR genes in this group. Group II consisted of 44 NLR genes that were induced by pathogens but not SA or BTH (Figure 3). All 131 NLR genes that were induced by pathogens or pathogen patterns were within Groups I and II, and 44 of them are not induced by SA or BTH, indicating a SA-independent induction by pathogens. Group III had 52 NLR genes including previously identified 29 non-expressed or “non-altered” NLR genes (Figure 3; Table 2). The transcripts of most NLR genes in this group were not altered in response to pathogen infection or SA and BTH treatment, except for two NLRs *AT1G72840* and *AT1G57850* showing inconsistent transcript changes in response to avirulent pathogens (Figure 3). Group IV contained 21 NLR genes repressed by pathogens, and 2 of them were repressed by BTH (Figure 3). These analyses indicate that biotic stresses had larger effects on NLR gene transcription and response to SA treatment is similar to responses to pathogens.

**Figure 4:**
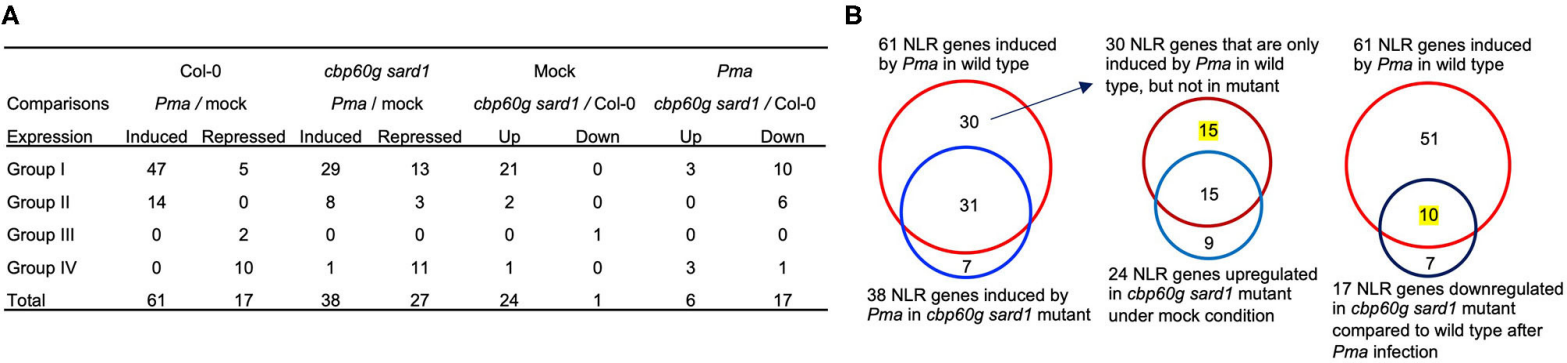
Number of NLR genes with altered expression in the *cbp60g sard1* double mutant. **(A)** Number of NLR DEGs in each group in the *cbp60g sard1* mutant and the wild type in response to *P. syringae* pv *maculicola* ES4326. DEGs were extracted from GSE10087 (Lu et al., 2018) by in-house pipeline with *p* < 0.05. “Up” and “Down” indicate increased or reduced expression in the mutant compared to the wild type, respectively. **(B)** Venn diagrams showing SA-dependent NLR gene induction by pathogen. The left panel is NLR genes induced by *Pma* in wild type and NLR genes in the *cbp60g sard1* double mutant. The middle panel is the NLR genes induced by *Pma* only in wild type and NLR genes upregulated in *cbp60g sard1* double mutant compared to the wild type under mock induction. The right panel shows the NLR genes induced by *Pma* in wild type and NLR genes downregulated in *cbp60g sard1* double mutant compared to wild type after *Pma* infection. The number of NLR genes whose pathogen induction or whose full induction are dependent on *CBP60G/SARD1* is highlighted with yellow. *Pma, P. syringae* pv *maculicola* ES4326.

In order to determine whether or not SA accumulation is responsible for induction of some NLRs during pathogen infection, we analyzed RNAseq data of the *cbp60g sard1* double mutant where SA induction by pathogen was greatly compromised (Lu et al., 2018). As expected, the six genes related to biosynthesis of SA and NHP, *PAD4, PBS3, ICS1, SARD4, ALD1*, and *FMO1* have reduced expression in the double mutant compared to the wild type after infection by *P. syringae* pv *maculicola* ES4326 (*Pma*) (Figure 3). With *p* < 0.05, a total of 61 NLRs were induced by *Pma* in the wild-type plants but only 38 NLRs were induced in the *cbp60g sard1* double mutant (Figure 4A). Among the 61 NLR genes induced by *Pma* in wild type, 31 NLR genes were also induced in the *cbp60g sard1* double mutant while 30 NLR genes were induced only in wild type, making them candidate NLRs whose induction by pathogen is dependent on SA induction (Figure 4B). Surprisingly, 15 out of these 30 NLR genes had an increased expression in the *cbp60g sard1* double mutant compared to the wild type under mock condition (Figure 4B). While it is yet to be determined why these NLRs, most of which can be induced by SA, had increased expression in the mutant deficient in SA induction, the higher expression under normal condition might contribute to their non-induction by *Pma* infection. The other 15 NLR genes were induced by *Pma* infection only in the wild type and did not have a higher expression in the mutant compared to the wild type under mock condition (Figures 3, 4B). The induction of these 15 NLR genes by pathogen in the wild type is dependent on *CBP60g*/*SARD1* and their mediated SA induction. In addition, 17 NLRs had reduced expression in the mutant compared to the wild type after *Pma* infection, and 10 of them were induced by *Pma* in the wild type (Figures 3, 4A). Therefore, the induction of these 10 NLRs in the wild type was compromised in the *cbp60g sard1* mutant. Two of these 10 NLRs were among the 15 NLR genes induced by *Pma* only in the wild type, indicating that 8 others had reduced induction by pathogen in the mutant compared to the wild type (Figure 3). These data indicate that the induction or full induction of a total of 23 (15 + 8) NLRs are dependent on *CBP60g* and *SARD1*, suggesting that their pathogen-induced expression is likely triggered by an increased SA production after pathogen infection.

### Genes in the Same Gene Cluster More Likely Have Similar Expression Patterns

We analyzed the expression patterns of NLR genes residing in the same gene cluster to determine if they were co-regulated. The Col-0 accession of *A. thaliana* has a total of 42 NLR gene clusters with 2–11 of NLR genes in one cluster (Meyers et al., 2003). Co-expression was defined as genes residing in the same or adjacent subclade in the Cluster analysis. Fifteen NLR gene clusters had at least two genes in the cluster showing co-expression (Figure 2). For example, *SOC3* (*AT1G17600*) and *CHS1* (*AT1G17610*) are two well-known NLR genes with TIR domain in the same gene cluster, and they were next to each other in the expression cluster subclade, indicating a similar expression pattern (Figure 2). Likewise, *SNC1, RECOGNITION OF PERONOSPORA PARASITICA 4* (*RPP4*), and *SIDEKICK SNC1 2* (*SIKIC2)* resided in the *RPP5* gene cluster, which contains eight NLR genes in the Col-0 background (Meyers et al., 2003), and they were clustered together in the expression cluster subclade (Figure 2). However, not all the genes in the same gene cluster shared a similar expression pattern. For instance, in the *RPP5* gene cluster, *SNC1, RPP4*, and *SIKIC2* were clustered together, while five other NLR genes in the *RPP5* gene cluster were in other modules (Figure 2). Co-regulation of NLR genes in the *RPP5* gene cluster has been implicated in previous studies (Yi and Richards, 2007; Zou et al., 2014, 2017). These results indicate that NLR genes in the same cluster are more likely to have a similar expression pattern, which might enable plants to initiate a timely and effective immune response.

### Limitations of This Meta-Analysis

Because this meta-analysis was from different datasets of separate studies, this raises an issue when comparing gene list across different studies. Biological differences and methodological differences could potentially make some cross comparisons impossible. These differences may come from (a) plant growth conditions (light quality and quantity; light cycle; humidity; growth medium); (b) developmental stage of plants; (c) tissues (leaf or whole plants) sampled for RNAseq analysis; (d) RNAseq method (library preparation; sequencing method and depth); and (e) RNAseq data analysis (methods and cutoffs for extracting DEGs). These factors can affect the DEG list extracted for analysis. The following measures have been used to minimize these differences: (1) Datasets from similar biological conditions were selected for this study. For instance, only leaves or young seedlings were selected (Supplementary Data 1); (2) When possible, datasets with processed reads count were selected so that the same in-house pipeline can be used to extract DEGs with the same selection criteria. In fact, DEGs from 66 out of the 88 RNAseq datasets were selected using the same criterion of *p* < 0.05. In addition, the selection criteria for the rest of the datasets (directly from the article) were mostly using the same selection criteria (*p* value, *q* value, or FDR < 0.05 with an additional FC ≥ 2). Characteristics that are not dependent on selection criteria or sample quality (such as more genes up than down) are used for cross-comparison between different studies. Characteristics that are dependent on selection methods are only compared within the same experimental set. Although we made efforts to minimize both biological and methodological differences, these factors need to be considered when it comes to cross-comparison of the number of NLR genes induced or repressed under stress conditions among different studies.

## Discussion

Plant NLR proteins are central intracellular immune receptors critical for pathogen recognition and immune response activation. They are known to be tightly regulated at the protein level for immunity/growth balance. However, their dynamics at the RNA transcript level was not extensively investigated before. This meta-analysis mined 88 RNAseq data and systematically revealed transcript dynamics of NLR genes during plant pathogen interaction and under abiotic stresses, which provides an extensive description of NLR expression under the changing environment.

### Transcriptional Induction of NLR Genes Is Prevalent in Plant Immune Response

The first striking feature of the meta-analysis is that more than half of NLR genes are induced by pathogens or defense elicitors and much fewer NLR genes were repressed than induced by biotic stresses (Table 1; Figures 1, 2). Additionally, more NLR genes were induced at later stage after pathogen infection, and avirulent pathogens and defense elicitors triggered NLR gene activation much faster compared to virulent pathogens (Table 1; Figure 1). Also, the number of NLR genes induced or repressed fluctuated throughout the duration of pathogen or defense elicitor treatments (Figure 1), indicating that the transcription of NLR genes is very dynamic in immune responses. Indeed, NLR genes need to be induced upon pathogen infection to confer disease resistance while the transcription of NLR genes also needs to be tightly monitored to prevent overactivation. For instance, most NLR genes upregulated in the *hos15-4* mutant are induced by pathogens while these NLRs are simultaneously repressed by HOS15 under pathogenic condition (Yang et al., 2020). These results suggest that regulation of NLR gene expression, in addition to the activation of NLR proteins, might be an important mechanism for plant to better fend off pathogen invasion.

### Transcriptional Repression of NLR Genes Might Be a Mechanism for Plant Adaption to Abiotic Stresses

The second feature is that the same set of NLR genes that are induced under biotic stress conditions are repressed by heat, ABA, and drought (Figure 2). These abiotic factors have been shown to inhibit disease resistance in general. For instance, the ABA biosynthetic loss-of-function mutant *aba3-1* is more resistant to *Pst* DC3000 and the ABA biosynthetic gain-of-function mutant *cds2-1D* exhibits susceptibility to various *P. syringae* strains as compared to wild type (Fan et al., 2009). The repression of plant defense response by ABA at least partially comes from its cross talk with SA (Mohr and Cahill, 2007; Yasuda et al., 2008; Fan et al., 2009). ABA treatment reduces SA concentration in plants and represses many genes involved in phenylpropanoid biosynthesis pathway, which is closely associated with SA biosynthesis (Mohr and Cahill, 2007). On the other hand, SA antagonizes ABA signaling through multiple mechanisms including inhibiting ABA-induced gene expression and acting against the role of ABA on protein degradation or stabilization (Manohar et al., 2017). Therefore, the opposite effects on NLR gene transcription by ABA and biotic stresses, which often induce SA accumulation, might be due to the antagonism between ABA and SA. In addition, plant drought response is largely through the regulation of ABA signaling pathways, which likely results in similar effects on NLR transcription by ABA and drought. Likewise, high temperature stimulates ABA biosynthesis (Toh et al., 2008), and it is possible that an ABA increase by heat stress contributes to the decrease of NLR transcript level at high temperature. Therefore, the induction of NLRs by biotic stresses and repression by abiotic stresses could result from antagonistic effects between SA and ABA. This study reveals that regulation of NLR gene expression might be an important node for balancing biotic and abiotic stress responses. It is not uncommon for a NLR gene to be functional in one natural accession but non-functional in another accession. Although this may result mainly from co-evolution between plants and pathogens, the balance between biotic and abiotic responses might also play a role. Indeed, functional and non-functional NLR gene *ACQUIRED OSMOTOLERANCE* (*ACQOS*) was maintained in Arabidopsis natural accessions due to trade-off between biotic and abiotic stress adaption (Ariga et al., 2017). The *ACQOS* gene was in module C, and it was generally induced under biotic stresses while repressed under abiotic stresses (Figure 2). Therefore, repression of NLR transcription might have evolved as a general mechanism for plant to survive under abiotic stresses.

### A Small Set of NLR Genes Are Repressed During Plant–Microbe Interaction

Interestingly, a small set of NLR genes are repressed by biotic stresses in contrast to the majority of NLR genes (Figure 2). Most of these genes are not functionally characterized (Figure 2). Expression suppression of some NLR genes may occur simultaneously with expression increase of other NLR genes in order to prevent overactivation of immune responses and its consequent fitness costs. For example, an immunity regulator ENHANCED DOWNY MILDEW 2 (EDM2) positively regulates expression of *RECOGNITION OF PERONOSPORA PARASITICA 7* (*RPP7*) and a small number of other NLR genes, whereas it represses the expression of a number of other NLR genes (Lai et al., 2020). Studying the molecular mechanism and biological relevance of NLR genes that are often repressed by pathogens in the context of plant–microbe interaction is expected to bring a new insight into NLR function.

### SA Contributes to NLR Induction Under Pathogenic Conditions

Co-expression of SA-related genes with many NLRs under biotic stresses suggests a role of SA in defense signaling transduction and amplification. SA induction was reported for several NLR genes and was postulated to amplify their function (Shirano et al., 2002; Xiao et al., 2003; Yang and Hua, 2004). This study revealed that the majority of NLR genes (in modules C and D) that were induced by pathogens were also induced by SA and co-expressed with SA-related genes (Figure 2). Conversely, SA and BTH induced expression of about 90 NLR genes, and almost all of them were also induced by pathogens (Figure 3). Induction of some of these NLRs by pathogen invasion happened before measurable SA induction, suggesting that SA might amplify their induction during infection. Analysis of SA-deficient mutant *cbp60g sard1* revealed that induction of 15 NLRs and full induction of the other 8 NLRs are dependent on *CBP60g* and *SARD1* and therefore likely SA accumulation. Therefore, SA is an inducer and an amplifier of some NLR genes. Additionally, NLR or NLR-like genes with TIR domain were more likely to be induced by SA (Figure 2). This is consistent with the previous finding that SA induced the expression of several TNL genes including *SSI4, RPP1*, and *RPS4*, but had little effect on the expression of two CNL genes *RPM1* and *RPS2* (Shirano et al., 2002). This might be due to a more prominent role of EDS1 and SA in TNL-mediated compared to CNL-mediated immune responses.

This analysis also identified 44 NLR genes whose expression are not significantly induced by SA or BTH. Their induction by pathogens is therefore likely SA independent. The transcriptional regulation of these NLR genes during pathogen infection awaits to be explored in the future. The systematic analyses of all NLR genes under various stress conditions in this study will be a useful resource for better understanding the correlation between SA and NLR induction during pathogen infection process.

### Co-regulation of NLR Genes in the Same Gene Cluster Is a Common Phenomenon

This study also revealed that co-expression of NLR genes in the same gene cluster is quite common during plant pathogen interaction. More than one-third of NLR gene clusters have co-expressed genes under stress conditions (Figure 2). This co-expression might be efficient in co-ordinate genes with similar functions as genes in the same cluster tend to have high sequence similarity or are functionally related (such as gene pair). Mechanisms for co-expression have been studied for the *RPP5* gene cluster. Chromatin-based gene regulation and RNA silencing have been postulated for achieving co-expression of multiple genes (Yi and Richards, 2007; Zou et al., 2014, 2017). An open or close chromatin structure initiated by transcriptional proteins influencing one NLR gene could cause chromatin structure changes in the gene cluster and thus affect the expression of neighboring genes. Alternatively, a common regulatory element (such as an enhancer) could be shared by NLR genes in the same gene cluster and thus enable co-expression. Co-regulation of NLR genes is expected to facilitate a timely and effective immune response upon pathogen infection.

## Conclusion

In sum, NLR genes have dynamic transcript expression patterns in response to both biotic and abiotic stresses. The majority of NLR genes are induced by biotic stresses and are repressed by heat and drought. The opposite effects from biotic and abiotic stresses suggest an important role of NLR gene expression in plant adaptation to environmental stresses. Plant hormones SA and ABA, respectively induce and repress NLR expression in general, suggesting a contribution of these two hormones to NLR gene regulation by biotic and abiotic stresses. Strikingly, a small set of NLR genes are repressed by both biotic and abiotic stresses, and their function will warrant further investigation. This study revealed a broad picture of dynamics of NLR transcript level under environmental stresses, which will facilitate a molecular understanding of immunity regulation under diverse stress conditions.

## Data Availability Statement

The original contributions presented in the study are included in the article/Supplementary Materials, further inquiries can be directed to the corresponding author/s.

## Author Contributions

JH and LY designed the study. LY and ZW performed the data analyses and made the figures and tables. LY and JH wrote the manuscript with input from ZW. All authors contributed to the article and approved the submitted version.

## Conflict of Interest

The authors declare that the research was conducted in the absence of any commercial or financial relationships that could be construed as a potential conflict of interest.
